# Starved Teeth

**Published:** 1891-10

**Authors:** 


### Starved Teeth.

Mr. J. Smith-Turner, in his presidential address to the British Dental Association recently, made an interesting statement with reference to decay in teeth. He said it often happened that though the sanitary conditions of the mouth were satisfactory, the teeth well formed and well placed, yet such teeth were susceptible of rapid destruction. There was to be seen in such a case the phenomena of a struggle for existence. The absence of the earthy salts was the cause of it. There was little difference between the teeth bone or dentine, and the bone of the frame The teeth were occupied in growing during the first twenty years, but the bones were likely to have the advantage in the competition. At the early period of life the bones of the skeleton were stimulated to growth by the activity of the young animal. There was much less activity in the teeth, and the same stimulus was absent, the supply of blood was small, and the same facility of nourishment was not afforded to them as in the bones of the skeleton. His object was to show that when the ordinary conditions of nutrition were disturbed, or the ordinary conditions absent, the teeth being slow in their appropriation of their share of nourishment were likely to suffer in their stability. Another element was the phosphorus in the brain. In any deficiency of the phosphates, the ever-active brain was more likely to appropriate its share of phosphates than the teeth. Hence there were three sources of danger; first, the competition of the osseous skeleton; second, the competition of the brain; and third, the comparatively small supply of blood to the teeth and the slowness of their development as terminal points in the circulation. These considerations rendered them very likely to suffer, and so to present the starved teeth so prevalent in the present day. Civilization had been blamed for much, but he was inclined to blame the liberties we took with Nature and her unerring processes for much of the dental distress now existing. With all the appearances of health and strength our teeth had been starved. The earthy salts must be taken into the body. There was no natural loboratory at work producing them and storing them up for the future. In other words, the great factors in earthy salts, so keenly competed for by other and more active parts of the body, must be supplied in our bodily diet. How far were such facts recognized and met? He did not say that a child might not flourish on other food than that originally intended for it by nature; but of this he was sure, and so was every medical practitioner, that the teeth of drugged children suffered terribly, and that whether the mother's deputy be a wet nurse or a feeding bottle, a great facility was afforded to those who were unscrupulous enough to disregard the future welfare of the child in taking their own ease. As we proceeded further we saw a continued disregard of the conditions he indicated. Children were now hurried through the earlier stages of their little lives, and manufactured into priggish little men and women before they had time to blush or weep. Children were being more and more sent from home for their education. If it must be so, he pleaded for some discrimination in their food. He could not believe that four hundred or five hundred boys could be fed on the same diet with benefit to all. He knew that this was recognized in some establishments; but in a general way, the barrack system prevailed. In feeding a regiment we were feeding men who had been chosen for certain physical qualities and certified health; but that was not the case with children. Home-made foods were now out of date, and whether it be from the exigencies of our dense population, or from impatience of natural processes, or from a combination of causes over which we might have control if we cared to try tinned meat, ready cooked and partly cooked preparations, and artificial foods were becoming more and more acceptable. All such things were prepared with a strict commercial view to price and to profit. They were all recommended as highly nutritious, and their facility of digestion was proved by quickly returning appetite. He feared that in many instances the quickly returned appetites showed that the true needs of the system had not been satisfied, and that the cheated organs were calling through the stomach for food, just as the dry throat and mouth expressed the need for water. The holidays of school children were a round of excitement, and at one period of the year a round of dissipation. Thus was the brain stimulated, and its activity assimilated even more than its share of the phosphates the circulation forms, and the teeth were starved.

*Exchange.*

